# Psychisch belastet: Wege in und durch die Arbeitswelt

**DOI:** 10.1007/s00103-024-03895-5

**Published:** 2024-07-08

**Authors:** Peter Angerer, Yesim Erim, Christoph Kröger, Joseph Kuhn, Eva Rothermund

**Affiliations:** 1 Institut für Arbeits‑, Sozial und Umweltmedizin, Heinrich-Heine-Universität Düsseldorf, Postfach 10 10 07, 40001 Düsseldorf, Deutschland; 2 Psychosomatische und Psychotherapeutische Abteilung, Friedrich-Alexander-Universität Erlangen-Nürnberg, Schwabachanlage 6, 91054 Erlangen, Deutschland; 3 Institut für Psychologie, Universität Hildesheim, Universitätsplatz 1, 31141 Hildesheim, Deutschland; 4 Bayerisches Landesamt für Gesundheit und Lebensmittelsicherheit, Veterinärstr. 2, 85764 Oberschleißheim, Deutschland; 5 Klinik für Psychosomatische Medizin und Psychotherapie, Universitätsklinikum Ulm, Albert-Einstein-Allee 23, 89081 Ulm, Deutschland

Das Themenheft des Bundesgesundheitsblattes mit dem Titel „Arbeiten mit psychischen Beeinträchtigungen: Neue Ansätze der Behandlung und Rehabilitation“ vereint verschiedene Blickwinkel aus Arbeitsmedizin, Psychosomatik, Psychotherapieforschung und Rehabilitationspsychologie auf die Versorgung von Arbeitnehmer:innen mit psychischen Störungen.

In den vergangenen 20 Jahren haben die Arbeitsunfähigkeits(AU)-Tage und Frühberentungen aufgrund von psychischen Störungen stetig zugenommen. Darüber hinaus zeigt sich bei Vorliegen einer psychischen Störung eine deutlich höhere Anzahl an AU-Tagen als bei vielen somatischen Erkrankungen. Neben volkswirtschaftlichen Auswirkungen wie Produktionsausfallkosten in Milliardenhöhe hat dies auch für den Einzelnen gravierende Folgen. So entfallen beispielsweise für die Betroffenen wichtige Funktionen von Arbeit, wie etwa soziale Anerkennung, die Anwendung von Kompetenzen und die Strukturierung des Alltags, welche die allgemeine Lebenszufriedenheit maßgeblich beeinflussen. Je länger eine Arbeitsunfähigkeit andauert, desto höher ist daher auch das Risiko einer Chronifizierung psychischer Störungen und einer Frühberentung. Des Weiteren erhöhen psychische Störungen das Risiko, arbeitslos zu werden, und vermindern die Chance, aus der Arbeitslosigkeit heraus wieder eine Arbeitstätigkeit zu finden.

Insofern besteht im Hinblick auf den Erhalt bzw. die Wiederherstellung der Arbeitsfähigkeit von Menschen mit psychischen Störungen erheblicher Handlungsbedarf. Dies gilt ungeachtet des Befunds aus großen epidemiologischen Studien, dass die Häufigkeit psychischer Störungen an sich – zumindest bis zur Coronakrise – nicht zugenommen hat. Vom Bundesgesundheitssurvey 1998 bis zur Studie zur Gesundheit Erwachsener in Deutschland (DEGS1) 2011 sind die Prävalenzen für psychische Störungen weitgehend unverändert geblieben. Etwa ein Drittel der Erwachsenen ist im Laufe eines Jahres von einer klinisch relevanten psychischen Störung betroffen, wenn man die im Bundesgesundheitssurvey 1998 erfassten Erkrankungen zugrunde legt. Neuere bevölkerungsrepräsentative Daten liegen nicht vor, wären aber dringend erforderlich, auch mit Blick auf die Folgen der Corona-Krise.

Durch die bessere Diagnostik und mehr Versorgungsangebote sowie eine partielle Enttabuisierung psychischer Störungen kommen mehr Menschen im Versorgungssystem an (siehe dazu auch Ausgabe 4/2023 – Public Mental Health). Das ist eine positive Entwicklung, allerdings mit Folgen. In den regelmäßig erscheinenden Krankenkassenreports wird immer wieder die hohe und teilweise steigende Anzahl an AU-Tagen infolge von psychischen Störungen berichtet. Gleiches gilt für die Entwicklung der krankheitsbedingten Frühberentungen infolge psychischer Störungen oder die Arzneimittelverordnungen. Hervorzuheben aus betrieblicher Sicht sind die mit AU-Tagen verbundenen Bruttowertschöpfungsverluste, Produktionsausfallkosten sowie die Lohnfortzahlungs- und Krankengeldzahlungen.

Die genannten Prävalenzzahlen zeigen, dass psychische Störungen auch in der Arbeitswelt kein Randphänomen sind, sondern viele Beschäftigte betreffen. Dabei ist es für die Betriebe in Zeiten des Fachkräftemangels auch personalwirtschaftlich sinnvoll, Beschäftigte mit einer psychischen Störung nicht zu verlieren, sondern im Betrieb zu halten und ggf. bei der Wiedereingliederung zu unterstützen. Die hierbei bestehenden Möglichkeiten sind aber oft gar nicht bekannt bzw. gelten zu Unrecht als unpraktikabel oder unfinanzierbar.

Die Arbeitswelt ist ein Ort, an dem viele Menschen ein Drittel des Tages und 40 Jahre ihres Lebens zubringen. Sie soll, wie die Ottawa-Charta der Weltgesundheitsorganisation einmal formulierte, eine Quelle der Gesundheit, nicht der Krankheit sein. Dazu gehören ganz wesentlich gesunde Arbeitsbedingungen und Maßnahmen der betrieblichen Gesundheitsförderung. Hier ist seit Inkrafttreten des Arbeitsschutzgesetzes 1996 viel passiert, die psychische Gesundheit gehört inzwischen zu den gängigen Handlungsfeldern der arbeitsweltbezogenen Prävention.

Gleichwohl gehören Menschen mit psychischen Störungen zum Alltag in den Betrieben, nicht anders als Menschen mit Rückenschmerzen oder Hauterkrankungen. Sie brauchen Hilfestellungen auf unterschiedlichen Ebenen. Im Rahmen dieses Themenheftes werden einerseits die Folgen von Arbeitsunfähigkeit bei psychisch Erkrankten auf Individual- und sozioökonomischer Ebene genauer betrachtet und andererseits aktuelle Befunde zu Therapie- und Rehabilitationsansätzen bei unterschiedlichen Beeinträchtigungen eingebracht. An dieser Stelle werden u. a. erste Ergebnisse zweier Verbundprojekte zu arbeitsbezogenen Interventionen – „Frühe Intervention am Arbeitsplatz“ (friaa) sowie „Blaufeuer“ – vorgestellt und diskutiert. Weitere Studien widmen sich der teilhabeorientierten psychotherapeutischen Behandlung. Darüber hinaus werden spezifische Belastungen von bestimmten Gruppen Erwerbstätiger verstärkt in den Blick genommen: Neben der Behandlung und Rehabilitation traumatisierter Erwerbstätiger wird dem Zusammenhang zwischen den Arbeitsbedingungen und der psychischen Gesundheit von Migrant:innen und Geflüchteten in Europa unter Berücksichtigung ihrer kulturellen Herkunft ein besonderes Augenmerk geschenkt.

Die vorliegende Ausgabe schließt damit auch wie angekündigt eine Lücke der erwähnten Ausgabe zu Public Mental Health. Wir danken den Autor:innen für ihre Beiträge und hoffen, die Leser:innen damit auf den einen oder anderen neuen Aspekt des Themas aufmerksam machen zu können – vielleicht auch hier und da in den Betrieben neue Ansätze der Unterstützung von Menschen mit psychischen Störungen anzuregen.

